# Oncological Safety of Diagnostic Hysteroscopy for Apparent Early-Stage Type II Endometrial Cancer: A Multicenter Retrospective Cohort Study

**DOI:** 10.3389/fonc.2022.918693

**Published:** 2022-06-23

**Authors:** Hui Zhou, Kai-Fa Lai, Qian Xiang, Yu Xu, Qian-Wen Zhang, Cui Hu, Xi-Guang Mao, Cheng Chen, Wu Huang, Gong-Sheng Mi, Juan Shen, Yong Tian, Feng-Mei Ke

**Affiliations:** ^1^ Department of Obstetrics and Gynecology, 363 Hospital, Chengdu, China; ^2^ Department of Obstetrics and Gynecology, Chengdu 363 Hospital Affiliated of Southwest Medical University, Chengdu, China; ^3^ Department of Obstetrics and Gynecology, West China Second University Hospital, Sichuan University, Chengdu, China; ^4^ Department of Obstetrics and Gynecology, The Affiliated Hospital of Southwest Medical University, Luzhou, China; ^5^ Department of Obstetrics and Gynecology, The Second Affiliated Hospital of Chengdu Medical College, Chengdu, China; ^6^ Department of Obstetrics and Gynecology, Mianyang Central Hospital, Mianyang, China; ^7^ Department of Obstetrics and Gynecology, Enshi Clinical College of Wuhan University, Enshi, China

**Keywords:** uterine serous carcinoma, uterine clear cell carcinoma, diagnostic hysteroscopy, overall survival, disease-free survival

## Abstract

**Objective:**

To study the oncological safety of diagnostic hysteroscopy for women with apparent early-stage type II endometrial cancer.

**Patients and Methods:**

A total of 429 women with presumed early-stage type II endometrial cancer were included. The 5-year disease-free survival (DFS) and overall survival (OS) were estimated and compared using the Kaplan-Meier method and the log-rank test among patients diagnosed by Dilation & Curettage (D&C) or diagnostic hysteroscopy. The Cox proportional hazards regression model was employed to adjust for potential confounding factors.

**Results:**

160 cases underwent D&C and 269 cases were diagnosed by diagnostic hysteroscopy. The 5-year DFS rate was 72.17% in the diagnostic hysteroscopy group and 76.16% in the D&C group, diagnostic hysteroscopy was not associated with deteriorated 5-year DFS rate (HR 1.25, 95% CI 0.84-1.86, *P*=0.281). The 5-year OS rate was 67.23% in the diagnostic hysteroscopy group and 70.71% in the D&C group, diagnostic hysteroscopy did not increase the risk of all-cause death (HR 1.11, 95% CI 0.78-1.57, *P*=0.573). Multivariable analysis showed that the method of endometrial sampling was not independently associated with DFS (aHR 1.38, 95% CI 0.92-2.07, *P*=0.122) and OS (aHR 1.23, 95% CI 0.85-1.77, *P*=0.272).

**Conclusion:**

For apparent early-stage type II endometrial cancer, endometrial sampling by diagnostic hysteroscopy was as safe as D&C.

## Introduction

In developed countries, endometrial cancer ranks first in common gynecological malignancies (1, 2). In 2020, endometrial cancer is diagnosed in about 420,000 women worldwide, and an estimated 98,000 women die from this cancer (3). To make matters worse, the incidence of endometrial cancer and the associated mortality are increasing among women of all backgrounds (2, 3).

In 1983, to reflect the disparate biologic behaviors and to refine the different prognoses, Bokhman classified endometrial cancer to type I cancers and type II cancers (4). Since then, this categorization system of endometrial cancer was universally adopted (2). Unlike type I endometrial cancer, type II endometrial cancer usually develops in nonobese women and is not related to hyperestrogenemia, endometrial hyperplasia, or metabolic syndrome (2, 5). Histologically, type II endometrial cancer is poorly differentiated or undifferentiated, including uterine serous carcinoma (USC), uterine clear cell carcinoma (UCCC), and uterine carcinosarcoma (2, 6, 7). Generally, type II endometrial cancer is clinically aggressive, usually presenting at advanced stages, having high rates of extrauterine involvement, and having a high risk of recurrence (2, 5, 6).

For women with endometrial cancer, the most common manifestation is abnormal uterine bleeding (2, 8). In women with abnormal uterine bleeding, to rule out malignant diseases, ultrasound and endometrial sampling are often required (8). Dilation & Curettage (D&C) and diagnostic hysteroscopy are the two most common methods for endometrial evaluation (8). Compared with D&C, by providing physicians with a visualization of the uterine cavity and facilitating the directed biopsy of suspicious lesions, diagnostic hysteroscopy is considered more accurate (9, 10). However, some researchers present their concerns. They think that in the process of diagnostic hysteroscopy, the elevated pressure in the uterine cavity may increase the risk of dissemination of cancer cells (11, 12). To date, however, many published studies have agreed that diagnostic hysteroscopy, although it can increase the spread of tumor cells into the peritoneal cavity, does not deteriorate the prognosis of endometrial cancer (13–16). But, it is worth noting that in these studies, almost all the included cases were low-risk endometrial cancer (14–16). Due to its rarity, the oncological safety of diagnostic hysteroscopy for type II endometrial cancers is always under-researched. Given the large biological and clinical heterogeneity between type I endometrial cancer and type II endometrial cancer, it is unknown whether diagnostic hysteroscopy is oncological safe for type II endometrial cancer.

Taken together, to explore the oncological safety of diagnostic hysteroscopy for apparent early-stage type II endometrial cancer, we conducted this multicenter retrospective cohort study.

## Patients and Methods

### Study Design

This was a multicenter retrospective cohort study, which was based on six Chinese teaching hospitals. This study was approved by the Institutional Review Board (IRB) of each participating institution. In consideration of the retrospective nature of the study design and this study did not report any identifiable private data, the written informed consent to participate was exempted by the IRBs of the participating centers.

### Patients

In this study, women with apparent early-stage type II endometrial cancer who had received a diagnosis during the 2011-2016 period and had been managed with surgical staging were included.

Patients would be eligible for this study if they met the following criteria: were between 18 and 80 years old, diagnosed with USC and UCCC by pathological examination, had no signs of a suspicious advanced disease, managed with surgical staging (at least including total hysterectomy, bilateral salpingo-oophorectomy, and pelvic lymphadenectomy) within one month after the definite diagnosis, and were consecutively followed up at the participating institutions.

In this study, the signs of suspected advanced diseases were defined as follows: suspicious involvement of the vagina, suspicious metastases of fallopian tubes and/or ovaries, enlarged regional lymph nodes (pelvic and/or para-aortic), or suspicious extrauterine metastases identified by pelvic examination or/and preoperative imaging (including ultrasound, computed tomography, and magnetic resonance imaging). All included cases were staged postoperatively based on the 2009 International Federation of Gynecology and Obstetrics (FIGO) staging system for endometrial cancer.

We excluded patients from this study for whom the method of endometrial sampling was unknown, those who lost to follow-up after initial management, those who were managed nonsurgically, those who had undergone neoadjuvant therapy, those who had a history of other malignancies, and those whose postoperative stage of disease was unknown. In this study, patients with a preoperative American Society of Anesthesiologists (ASA) physical status score of IV or larger were also considered not qualified for inclusion.

### Data Collection

Demographic, clinical, and pathological data of the included cases were extracted from the medical record management systems of the participating institutions. The data of interest were as follows: year of diagnosis, age at diagnosis, marital status at diagnosis, body mass index (BMI) at diagnosis, the preoperative ASA physical status score, the histological type of the tumor, the grade of tumor differentiation, tumor size, the FIGO stage of disease, the status of lymphovascular space invasion (LVSI), the result of peritoneal cytology, the approach of surgical staging, the scope of lymphadenectomy, the method of endometrial sampling, and the protocol of postoperative adjuvant therapy. Given the retrospective nature of this study, we accepted the clinical heterogeneity in the method of performing diagnostic hysteroscopy among the participating institutions, such as the pressure value of the solution jet, the number of biopsies, and the place of diagnostic hysteroscopy (an office setting or operative room setting), etc.

### Outcomes of Interest

In this study, disease-free survival (DFS) and overall survival (OS) were the primary outcomes of interest. DFS was defined as the time from diagnosis to disease recurrence or death from endometrial cancer. OS was defined as the time from diagnosis to death from any cause.

All included patients were followed up to death or until January 1, 2022. Data regarding patients with no evidence of recurrence or death were censored at the date of the last follow-up. Data on survival outcomes were collected as follows: vital status, time of disease recurrence, site of disease recurrence, time of death, and cause of death.

### Statistical Analysis

Based on the method of endometrial sampling, the included patients were divided into the D&C group and the diagnostic hysteroscopy group. The baseline characteristics were compared between the two groups. When assumptions of normal distribution were confirmed, comparisons of continuous variables would be performed by parametric methods. While the comparisons of non-normally distributed variables and categorical data were performed using nonparametric tests.

The Kaplan–Meier method was employed to generate the survival curves. The comparisons of the survival outcomes between the D&C group and the diagnostic hysteroscopy group were carried out by using the Log-rank test. To adjust the unbalanced confounding factors between the two groups, the Cox proportional hazards regression model was employed to estimate the adjusted hazard ratios (aHR) and 95% confidence intervals (95% CI) for the effect of diagnosis methods on DFS and OS in women with apparent early-stage type II endometrial cancer. To ensure parsimony of the final model, the following variables would be included in the Cox proportional hazards regression model: that was considered clinically relevant to prognosis or that showed a univariate relationship (*P*-value < 0.2) with outcomes of interest.

Statistical analyses were performed using STATA software, version 17 (StataCorp). Unless otherwise stated, all analyses were carried out with a two-sided significance level of 0.05.

## Results

### Study Cohort

**Figure 1:**
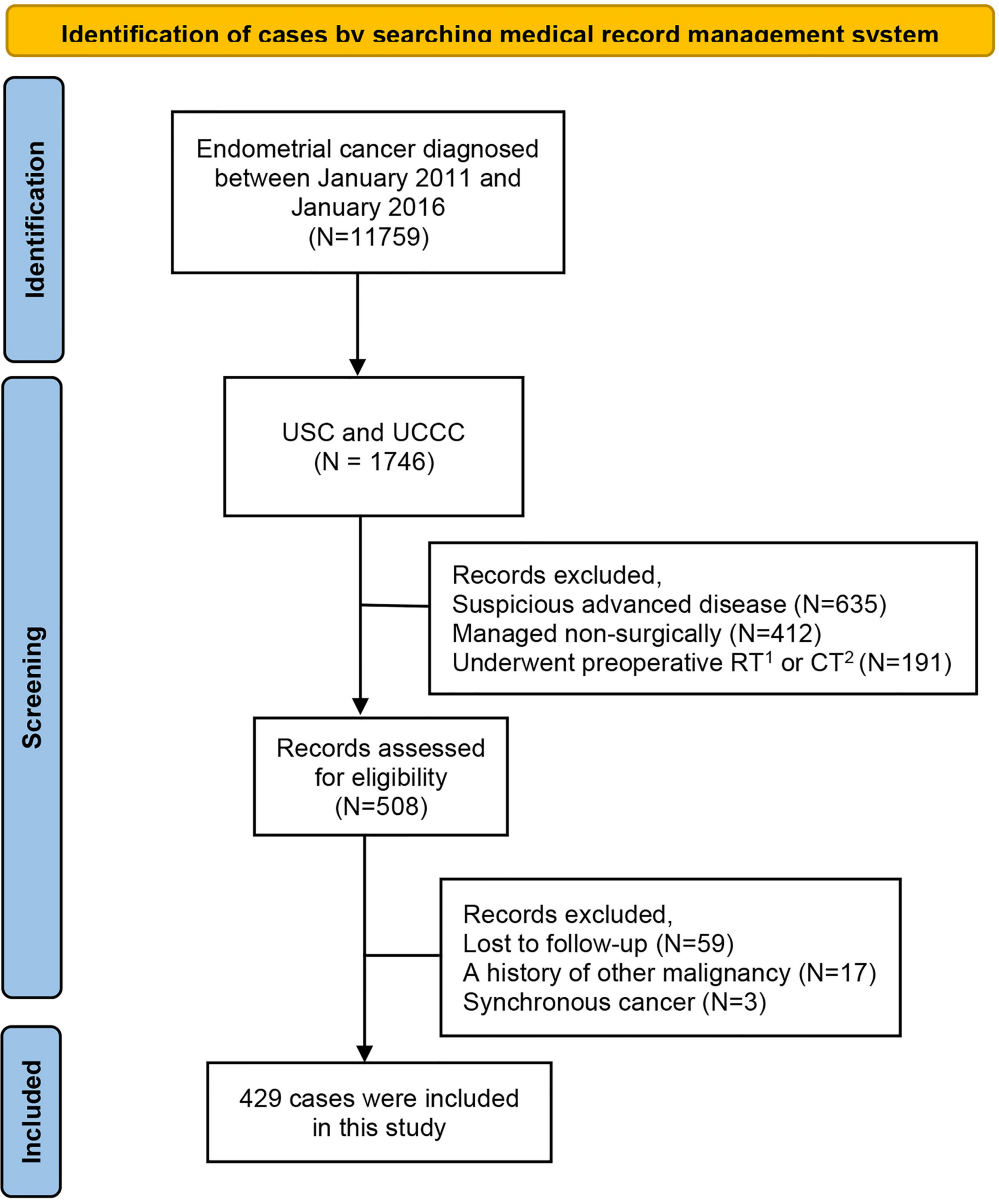
Flow chart of cases selection.

Between January 2011 and January 2016, a total of 11,759 women with endometrial cancer were managed at these participating institutions. After excluding 11,330 patients who were not qualified for the current study, a total of 429 women with apparent early-stage type II endometrial cancer were included in this study. The process of case selection is presented in **Figure 1**. Among the included patients, 160 patients (37.3%) got diagnosed by diagnostic hysteroscopy, the remaining patients were diagnosed by D&C.

According to the methods of endometrial sampling, the included patients were divided into the D&C group (N=269) and the diagnostic hysteroscopy group (N=160). The comparisons of patient demographics, clinicopathologic characteristics, and treatment variables between the D&C group and the diagnostic hysteroscopy group are summarized in **Table 1**.

**Table 1:** Characteristics of the study cohort^a^.

	Overall	Dilation & Curettage group	Hysteroscopy group	*P*
Year of diagnosis				0.172
2011-2013	147 (34.3%)	99 (36.8%)	48 (30.0%)	
2014-2016	282 (65.7%)	170 (63.2%)	112 (70.0%)	
Age at diagnosis (year)	66.5 ± 7.62	66.9 ± 7.72	65.8 ± 7.41	0.171
Duration of follow-up (month)	50 (4-107)	52 (4-107)	44.5 (4-107)	0.071
Marital status at diagnosis				0.003
Married	223 (52.0%)	155 (57.6%)	68 (42.5%)	
Single^b^	206 (48.0%)	114 (42.4%)	92 (57.5%)	
Body mass index	22.6 ± 4.08	22.5 ± 3.97	22.7 ± 4.27	0.654
ASA^c^ score				0.236
I	265 (61.8%)	173 (64.3%)	92 (57.5%)	
II	87 (20.3%)	48 (17.8%)	39 (24.4%)	
III	77 (17.9%)	48 (17.8%)	29 (18.1%)	
Histology				0.059
Clear cell carcinoma	120 (28.0%)	84 (31.2%)	36 (22.5%)	
Serous carcinoma	309 (72.0%)	185 (68.8%)	124 (77.5%)	
Grade				>0.999
Poorly differentiated	274 (63.9%)	172 (63.9%)	102 (63.8%)	
Undifferentiated	155 (36.1%)	97 (36.1%)	58 (36.2%)	
Tumor size				0.023
Less than 4cm	272 (63.4%)	182 (67.7%)	90 (56.2%)	
At least 4cm	157 (36.6%)	87 (32.3%)	70 (43.8%)	
Postoperative stage^d^				0.091
I/II	344 (80.2%)	215 (79.9%)	129 (80.6%)	
III/IV	85 (19.8%)	54 (20.1%)	31 (19.4%)	
LVSI^e^				0.363
Negative	319 (74.4%)	204 (75.8%)	115 (71.9%)	
Positive	110 (25.6%)	65 (24.2%)	45 (28.1%)	
Peritoneal cytology				<0.001
Negative	341 (79.5%)	232 (86.2%)	109 (68.1%)	
Positive	88 (20.5%)	37 (13.8%)	51 (31.9%)	
Approach of staging				0.537
Laparoscopy	267 (62.2%)	164 (61.0%)	103 (64.4%)	
Laparotomy	162 (37.8%)	105 (39.0%)	57 (35.6%)	
Lymphadenectomy				0.606
Pelvic	272 (63.4%)	168 (62.5%)	104 (65.0%)	
Pelvic and para-aortic	157 (36.6%)	101 (37.5%)	56 (35.0%)	
Adjuvant therapy				0.760
No	109 (25.4%)	66 (24.5%)	43 (26.9%)	
RT^f^ or CT^g^	191 (44.5%)	119 (44.2%)	72 (45.0%)	
Combined RT and CT	129 (30.1%)	84 (31.2%)	45 (28.1%)	

a Values are presented as mean ± standard deviation, median (minimum–maximum), or as number (percentage).

b Includes divorced, widowed, separated, and never married.

c The American Society of Anesthesiologists.

d Based on the 2009 staging system of the International Federation of Gynecology and Obstetrics.

e Lymphovascular space invasion.

f Radiotherapy.

g Chemotherapy.

For the entire cohort, the mean age was 66.5 years (standard deviation: 7.62), and the median duration of follow-up was 50 months (range: 4 months to 107 months). In terms of the age at diagnosis and the duration of follow-up, there was no statistical difference between the two groups (*P*=0.171 and *P*=0.071, respectively). There was also no statistical difference in the mean BMI between the two groups, 22.5 kg/m^2^ and 22.7 kg/m^2^, respectively. At diagnosis, the proportion of patients being single (including divorced, widowed, separated, and never married) in the hysteroscopy group was significantly higher than that in the D&C group (*P*=0.003).

In terms of the clinicopathological features of the tumors, 72% of cases were serous carcinomas, about 64% of tumors were poorly differentiated and less than 4 cm, about 20% of cases were found to be advanced (FIGO stage III or IV), 20.5% of patients were identified with positive peritoneal cytology, and 25.6% of the included patients had LVSI. Generally, the histologic type, the grade of tumor differentiation, the size of the tumor, the stage of the disease, and the incidence of LVSI were statistically similar between the D&C group and the diagnostic hysteroscopy group. However, the proportion of patients with positive peritoneal cytology in the diagnostic hysteroscopy group was significantly higher than that in the D&C group, at 31.9% and 13.8%, respectively. The difference in the incidence of positive peritoneal cytology between the two groups was statistically significant (*P<* 0.001).

Of the included patients, 62.2% got surgical staged by laparoscopy, 36.6% underwent complete regional lymph node removal (combined pelvic and para-aortic lymphadenectomy), and about 75% had postoperative adjuvant therapy (chemotherapy or/and radiotherapy). The protocol of management (surgical approach of staging, extent of lymphadenectomy, and postoperative adjuvant therapy) between the D&C group and the hysteroscopy group was not statistically different.

### Survival Outcomes

A total of 55 patients experienced disease recurrence, 18 from the diagnostic hysteroscopy group and the rest from the D&C group, rates of disease recurrence were not statistically different between the two groups (*P*=0.551). In terms of the pattern of disease recurrence in the two groups, the three most common sites of recurrence are the abdomen (3.0%), lungs (2.6%), and pelvis (1.9%). There was no statistical difference in the pattern of disease recurrence between the two groups (*P>*0.999). **Table 2** shows the pattern and rate of disease recurrence by diagnostic hysteroscopy vs. D&C.

**Table 2:** Patterns and rates of disease recurrence by diagnostic hysteroscopy vs. Dilation & Curettage.

	Diagnostic hysteroscopy group	Dilation & Curettage group	*P*
	(N=160)	(N=269)	
Disease recurrence			0.551
No	142 (88.8%)	232 (86.2%)	
Yes	18 (11.2%)	37 (13.8%)	
Site of recurrence			> 0.999
Vagina	2 (1.3%)	3 (1.1%)	
Pelvis	2 (1.3%)	6 (2.2%)	
Abdomen	4 (2.5%)	9 (3.3%)	
Nodal	2 (1.3%)	3 (1.1%)	
Liver	2 (1.3%)	4 (1.5%)	
Lung	4 (2.5%)	7 (2.6%)	
Bone	1 (0.6%)	2 (0.7%)	
Multiple	1 (0.6%)	3 (1.1%)	

**Figure 2:**
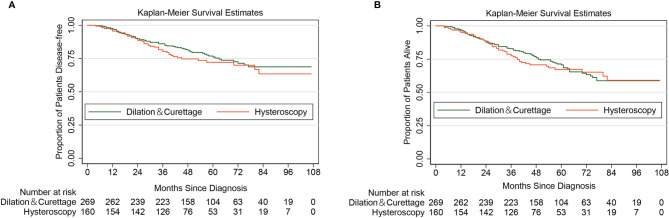
Kaplan-Meier curves of disease-free survival and overall survival for patients with apparent early-stage type II endometrial cancer, by the methods of endometrial sampling. (**A** for disease-free survival; **B** for overall survival).

With a median follow-up of 50 months, a total of 106 cases of recurrence or/and death from endometrial cancer were identified. **Supplementary Material 1A** shows the DFS curve of the entire cohort. Among them, 63 cases were from the D&C group, and the remaining 43 cases were in the diagnostic hysteroscopy group. The 5-year DFS rate by the Kaplan-Meier method was 72.17% (95% CI 63.68%–79.00%) in the diagnostic hysteroscopy group and 76.16% (95% CI 69.91%–81.29%) in the D&C group. The Log-rank test indicated that for patients with apparent early-stage type II endometrial cancer, diagnostic hysteroscopy was not associated with deteriorated 5-year DFS (HR 1.25, 95% CI 0.84-1.86, *P*=0.281). **Figure 2A** shows the Kaplan-Maier curve of DFS (diagnostic hysteroscopy *VS.* D&C).

As of January 1, 2022, a total of 135 all-cause deaths have been confirmed. **Supplementary Material 1B** shows the OS curve of the entire cohort. Among them, 84 cases were from the D&C group, and the remaining 51 cases were in the diagnostic hysteroscopy group. The 5-year OS rate by the Kaplan-Meier method was 67.23% (95% CI 58.60%–74.45%) in the diagnostic hysteroscopy group and 70.71% (95% CI 64.30%–76.18%) in the D&C group. For women with apparent early-stage type II endometrial cancer, diagnostic hysteroscopy did not increase the risk of all-cause death (HR 1.11, 95% CI 0.78-1.57, *P*=0.573). **Figure 2B** shows the Kaplan-Maier curve of OS (diagnostic hysteroscopy *VS.* D&C).

**Figure 3:**
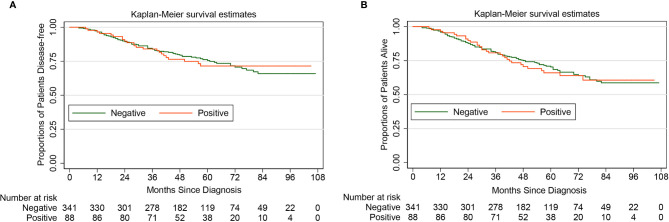
Kaplan-Meier curves of disease-free survival and overall survival for patients with apparent early-stage type II endometrial cancer, by peritoneal cytology. (**A** for disease-free survival; **B** for overall survival).

Theoretically, diagnostic hysteroscopy can increase the risk of tumor cells spreading into the peritoneal cavity, this was consistent with the finding of our study (**Table 1**). However, the Kaplan-Meier method and the Log-rank test showed that for women with apparent early-stage type II endometrial cancer, the positive peritoneal cytology was not associated with the deterioration of DFS (HR 1.03, 95% CI 0.65-1.64, *P*=0.901) and OS (HR 1.06, 95% CI 0.70-1.60, *P*=0.797). **Figure 3** shows the Kaplan-Maier curves of DFS and OS (positive peritoneal cytology *VS.* negative peritoneal cytology).

### The Cox Proportional Hazards Regression Analysis of Survival in Patients With Apparent Early-Stage Type II Endometrial Cancer

Based on the results of univariate analysis (**Supplementary Material 2**) and considering the clinical relevance of the candidate variables, the following variables were included in the Cox proportional hazards regression model: age at diagnosis, BMI at diagnosis, the preoperative ASA physical status score, tumor size, the postoperative FIGO stage of the disease, the status of LVSI, adjuvant therapy, and the method of endometrial sampling. The results of the Cox proportional hazards regression analysis demonstrated that for women with apparent early-stage type II endometrial cancer, the methods of preoperative endometrial sampling did not affect the oncological survival (for DFS: diagnostic hysteroscopy *VS.* D&C, aHR 1.38, 95% CI 0.92-2.07, *P*=0.122; for OS: diagnostic hysteroscopy *VS.* D&C, aHR 1.23, 95% CI 0.85-1.77, *P*=0.272).

The Cox proportional hazards regression model also indicated that for apparent early-stage type II endometrial cancer, having a preoperative ASA physical status score of III (III *VS.* I: aHR 2.11, 95% CI 1.01-4.43, *P*=0.048), having an advanced disease (III/IV *VS.* I/II: aHR 2.68, 95% CI 1.68-4.28, *P*=0.000), and having LVSI (Yes *VS.* No: aHR 2.71, 95% CI 1.49-4.95, *P*=0.001) could worsen the DFS of patients; while postoperative adjuvant therapy was beneficial to the DFS of patients (radiotherapy or chemotherapy *VS.* without adjuvant therapy: aHR 0.54, 95% CI 0.34-0.87, *P*=0.011; combined radiotherapy and chemotherapy *VS.* without adjuvant therapy: aHR 0.39, 95% CI 0.23-0.67, *P*=0.001). In terms of the risk of all-cause death in patients with apparent early-stage type II endometrial cancer, age at diagnosis (*P*=0.039), the preoperative ASA physical status score (*P*=0.029), the stage of disease (*P*=0.000), the status of LVSI (*P*=0.000), and postoperative adjuvant therapy (*P*=0.000) were all independent predictors. **Table 3** shows the Cox proportional hazards regression model for survival in patients with apparent early-stage type II endometrial cancer.

**Table 3:** Multivariate analysis of prognosis for women with apparent early-stage type II endometrial cancer.

	DFS^a^			OS^b^		
	aHR^c^	95% CI^d^	*P*	aHR	95% CI	*P*
Age						
< 65 years	Reference			Reference		
≥ 65 years	1.34	0.82-2.17	0.239	1.58	1.02-2.43	0.039
Body mass index						
< 24 kg/m^2^	Reference			Reference		
≥ 24 kg/m^2^	1.19	0.74-1.91	0.470	1.21	0.79-1.85	0.391
ASA^e^ score			0.035			0.029
I	Reference			Reference		
II	1.18	0.544-2.56	0.676	1.18	0.46-1.88	0.138
III	2.11	1.01-4.43	0.048	2.33	1.16-3.65	0.025
Tumor size						
< 4 cm	Reference			Reference		
≥ 4 cm	1.36	0.82-2.28	0.237	1.17	0.740-1.85	0.502
Stage (FIGO^f^ 2009)						
I/II	Reference			Reference		
III/IV	2.68	1.68-4.28	0.000	3.08	2.01-4.71	0.000
LVSI^g^						
Negative	Reference			Reference		
Positive	2.71	1.49-4.95	0.001	2.80	1.60-4.88	0.000
Adjuvant therapy			0.002			0.000
No	Reference			Reference		
RT^h^ or CT^i^	0.54	0.34-0.87	0.011	0.47	0.31-0.71	0.000
RT and CT	0.39	0.23-0.67	0.001	0.34	0.21-0.55	0.000
Method of diagnosis						
Dilation&Curettage	Reference			Reference		
Hysteroscopy	1.38	0.92-2.07	0.122	1.23	0.85-1.77	0.272

a Disease-free survival.

b Overall survival.

c adjusted hazard ratio.

d Confidence interval.

e American Society of Anesthesiologists.

f The International Federation of Gynecology and Obstetrics.

g Lymphovascular space invasion.

h Radiotherapy.

i Chemotherapy.

## Discussion

Based on six Chinese tertiary hospitals, this multicenter retrospective cohort study finds that for women with apparent early-stage type II endometrial cancer, diagnostic hysteroscopy was as safe as traditional D&C.

Postmenopausal bleeding, unscheduled bleeding, and menorrhagia are very common gynecologic complaints (17, 18). The main purpose of the management for these women is to rule out malignant lesions or diseases with malignant potentials, such as cancer of the endometrium and endometrial hyperplasia (19). For the elderly with abnormal uterine bleeding, all kinds of evaluations are justified by the common acceptance that postmenopausal bleeding is “cancer until proven otherwise” (20). Thus, for women with abnormal uterine bleeding, the necessity of endometrial sampling is mainly based on the risk of endometrial cancer (20, 21).

The sensitivity of endometrial sampling is high for the identification of endometrial lesions (endometrial cancer included), and D&C has been the standard procedure for diagnosing cancer of the endometrium for years (22). However, with the advances in instrumentation, hysteroscopy plays an increasingly important role in the diagnosis of endometrial cancer, even in an ambulatory setting (23, 24). With endoscopic visualization of the endometrial cavity and the directed biopsy, diagnostic hysteroscopy is considered more accurate and reliable than traditional D&C in diagnosing endometrial lesions (9, 25, 26). A meta-analysis conducted by Bourdel et al. found that for patients with atypical endometrial hyperplasia, compared with D&C, diagnostic hysteroscopy results in a lower underestimation of endometrial cancer (27). However, the high pressure of the uterine cavity during the process of hysteroscopy may facilitate the spreading of tumor cells into the abdominal cavity. Having 1015 women with endometrial cancer included, the study by Polyzos et al. reported that compared with patients who did not undergo diagnostic hysteroscopy, those who underwent diagnostic hysteroscopy had a significantly higher rate of malignant peritoneal cytology (odds ratio 1.78, 95% CI 1.13-2.79, *P*=0.013) *(* 28). This finding was consistent with that of many other studies (11, 29, 30). In our study, the rate of positive peritoneal cytology in the diagnostic hysteroscopy group was also significantly higher than that in the D&C group, 31.9% and 13.8%, respectively.

But, the negative effects of tumor cells disseminated into the peritoneal cavity during diagnostic hysteroscopy on the prognosis of women with endometrial cancer are not well established. Although the result of peritoneal cytology is no longer a factor to consider in the 2009 FIGO staging system for endometrial cancer, numerous studies still find that malignant peritoneal cytology is strongly associated with the deterioration of long-term prognosis in patients with endometrial cancer (31–34). However, some facts deserve our attention. Almost all of the included cases in the mentioned studies were endometrioid adenocarcinoma of the endometrium (31–34). Few studies have reported the prognostic significance of malignant peritoneal cytology in type II endometrial cancer. What is more, all the malignant peritoneal cytology in the mentioned studies was not associated with diagnostic hysteroscopy (31–34). Whether the malignant cells disseminated into the peritoneal cavity during diagnostic hysteroscopy can survive, colonize, invade the normal tissue, and worsen the prognosis of patients is unknown. A systematic review and meta-analysis by Du et al. showed that for endometrial cancer, although can increase the risk of spreading of malignant cells, diagnostic hysteroscopy did not worsen the prognosis (13). With 127 type II endometrial cancer cases included, the study conducted by Ribeiro et al. also reported that compared with traditional D&C, diagnostic hysteroscopy did not increase the risk of recurrence and all-cause death (35). This result is consistent with ours. But, large and adequately powered prospective studies with long-term follow-up are still needed to testify the safety of diagnostic hysteroscopy for type II endometrial cancer. Until such studies become available, we still need to be careful about the employment of diagnostic hysteroscopy in type II endometrial cancer.

Based on six centers, our study has a sample size of 429 patients. Considering the rarity of type II endometrial cancer, the sample size of the current study is relatively large. Also, the entire cohort underwent a long-term follow-up. However, there are some limitations to our study. First, due to the limited resources, the pathological diagnoses of UCCC and USC were not reviewed again by experts in pathology. We extracted postoperative pathological diagnoses from patients’ electronic medical records. Second, the pressure of the uterine cavity during diagnostic hysteroscopy was not reported in patients’ electronic medical records. Therefore, we could not explore the effect of intrauterine pressure during diagnostic hysteroscopy on the long-term survival of type II endometrial cancer patients who underwent diagnostic hysteroscopy. Third, there was possible confounding by indications of diagnostic hysteroscopy due to our study design. In clinical practice, there is currently no widely accepted indication for diagnostic hysteroscopy in the diagnosis of endometrial cancer. Gynecologists of the participating centers of this study chose the method of endometrial sampling mainly based on their preference and judgment. The last, considering the retrospective nature of the current study, there were some inevitable biases, such as recall bias, selection bias, etc. To reduce these biases as much as possible, we screened cases strictly according to established inclusion and exclusion criteria and excluded those with incomplete data.

## Conclusion

For apparent early-stage type II endometrial cancer, endometrial sampling by diagnostic hysteroscopy is as safe as traditional D&C. This finding needs further large and adequately powered prospective studies to verify.

## Data Availability Statement

The raw data supporting the conclusions of this article will be made available by the authors, without undue reservation.

## Ethics Statement

Ethical review and approval was not required for the study on human participants in accordance with the local legislation and institutional requirements. Written informed consent for participation was not required for this study in accordance with the national legislation and the institutional requirements.

## Author Contributions

HZ: Conceptualization, Methodology, Investigation, Writing – original draft, Writing – review & editing, and Supervision. YX: Methodology, Investigation, Data analysis, Writing – original draft, Writing – review & editing, and Project administration. K-FL, QX, Q-WZ, CH, X-GM, CC, WH, G-SM, JS, YT, and F-MK: Methodology, Investigation, Data analysis, and Writing – review & editing. All authors contributed to the article and approved the submitted version.

## Conflict of Interest

The authors declare that the research was conducted in the absence of any commercial or financial relationships that could be construed as a potential conflict of interest.

## Publisher’s Note

All claims expressed in this article are solely those of the authors and do not necessarily represent those of their affiliated organizations, or those of the publisher, the editors and the reviewers. Any product that may be evaluated in this article, or claim that may be made by its manufacturer, is not guaranteed or endorsed by the publisher.

## Supplementary Material

Supplementary Material 1 Kaplan-Meier curves of disease-free survival and overall survival for patients with apparent early-stage type II endometrial cancer. (**A** for disease-free survival; **B** for overall survival).

Supplementary Material 2 Univariate analysis of prognosis for women with apparent early-stage type II endometrial cancer.
