# Supplementary material for: Oncological Safety of Diagnostic Hysteroscopy for Apparent Early-Stage Type II Endometrial Cancer: A Multicenter Retrospective Cohort Study
Source: Front Oncol. 2022 Jun 23;12:918693. doi: 10.3389/fonc.2022.918693 (PMC9259840; doi:10.3389/fonc.2022.918693)
Supplement: Supplementary Material 1 — Kaplan-Meier curves of disease-free survival and overall survival for patients with apparent early-stage type II endometrial cancer. (A for disease-free survival; B for overall survival). [file DataSheet_1.zip › Supplementary Material 2.docx]

Supplementary material 2. Univariate analysis of prognosis for women with apparent early-stage type Ⅱ endometrial cancer

|  | DFS^a^ | | |  | OS^b^ | | |
| --- | --- | --- | --- | --- | --- | --- | --- |
|  | HR^c^ | 95% CI^d^ | *P* |  | HR | 95% CI | *P* |
| Age |  |  |  |  |  |  |  |
| < 65 years | Reference |  |  |  | Reference |  |  |
| ≥ 65 years | 1.89 | 1.29-2.79 | 0.003 |  | 2.12 | 1.50-2.98 | 0.000 |
| Marital status |  |  |  |  |  |  |  |
| Married | Reference |  |  |  | Reference |  |  |
| Single^e^ | 1.39 | 0.86-2.87 | 0.591 |  | 1.18 | 0.55-1.96 | 0.681 |
| Body mass index |  |  |  |  |  |  |  |
| < 24 kg/m^2^ | Reference |  |  |  | Reference |  |  |
| ≥ 24 kg/m^2^ | 1.44 | 1.07-2.36 | 0.011 |  | 1.34 | 1.18-2.08 | 0.016 |
| ASA^f^ score |  |  |  |  |  |  |  |
| Ⅰ | Reference |  |  |  | Reference |  |  |
| Ⅱ | 2.11 | 1.17-3.82 | 0.003 |  | 1.82 | 1.10-3.01 | 0.007 |
| Ⅲ | 5.30 | 2.99-9.41 | 0.000 |  | 4.10 | 2.47-6.82 | 0.000 |
| Histology |  |  |  |  |  |  |  |
| Serous carcinoma | Reference |  |  |  | Reference |  |  |
| Clear cell cancer | 1.05 | 0.69-1.60 | 0.824 |  | 1.05 | 0.72-1.53 | 0.801 |
| Grade |  |  |  |  |  |  |  |
| Poorly differentiated | Reference |  |  |  | Reference |  |  |
| Undifferentiated | 1.13 | 0.75-1.69 | 0.542 |  | 1.17 | 0.81-1.67 | 0.387 |
| Tumor size |  |  |  |  |  |  |  |
| < 4 cm | Reference |  |  |  | Reference |  |  |
| ≥ 4 cm | 1.99 | 1.33-2.98 | 0.000 |  | 1.92 | 1.34-2.74 | 0.000 |
| Stage (FIGO^g^ 2009) |  |  |  |  |  |  |  |
| Ⅰ/Ⅱ | Reference |  |  |  | Reference |  |  |
| Ⅲ/Ⅳ | 4.01 | 2.39-6.73 | 0.000 |  | 3.87 | 2.44-6.14 | 0.000 |
| LVSI^h^ |  |  |  |  |  |  |  |
| Negative | Reference |  |  |  | Reference |  |  |
| Positive | 3.39 | 2.12-5.42 | 0.000 |  | 2.98 | 1.96-4.53 | 0.000 |
| Peritoneal cytology |  |  |  |  |  |  |  |
| Negative | Reference |  |  |  | Reference |  |  |
| Positive | 1.03 | 0.65-1.64 | 0.901 |  | 1.06 | 0.70-1.60 | 0.797 |
| Approach of staging |  |  |  |  |  |  |  |
| Laparotomy | Reference |  |  |  | Reference |  |  |
| Laparoscopy | 1.04 | 0.70-1.54 | 0.840 |  | 1.11 | 0.79-1.58 | 0.544 |
| Lymphadenectomy |  |  |  |  |  |  |  |
| Pelvic | Reference |  |  |  | Reference |  |  |
| Pelvic plus para-aortic | 0.99 | 0.67-1.48 | 0.991 |  | 0.89 | 0.63-1.26 | 0.508 |
| Adjuvant therapy |  |  |  |  |  |  |  |
| No | Reference |  |  |  | Reference |  |  |
| RT^i^ or CT^j^ | 0.67 | 0.42-0.99 | 0.041 |  | 0.56 | 0.37-0.86 | 0.003 |
| Combined RT and CT | 0.64 | 0.38-0.88 | 0.004 |  | 0.44 | 0.28-0.70 | 0.000 |

^a^Disease-free survival

^b^Overall survival

^c^Hazard ratio

^d^Confidence interval

^e^Includes divorced, widowed, separated, and never married

^f^The American Society of Anesthesiologists

^g^The International Federation of Gynecology and Obstetrics

^h^Lymphovascular space invasion

^i^Radiotherapy

^j^Chemotherapy
