# Supplementary figures and images for: Oncological Safety of Diagnostic Hysteroscopy for Apparent Early-Stage Type II Endometrial Cancer: A Multicenter Retrospective Cohort Study
Source: Front Oncol. 2022 Jun 23;12:918693. doi: 10.3389/fonc.2022.918693 (PMC9259840; doi:10.3389/fonc.2022.918693)

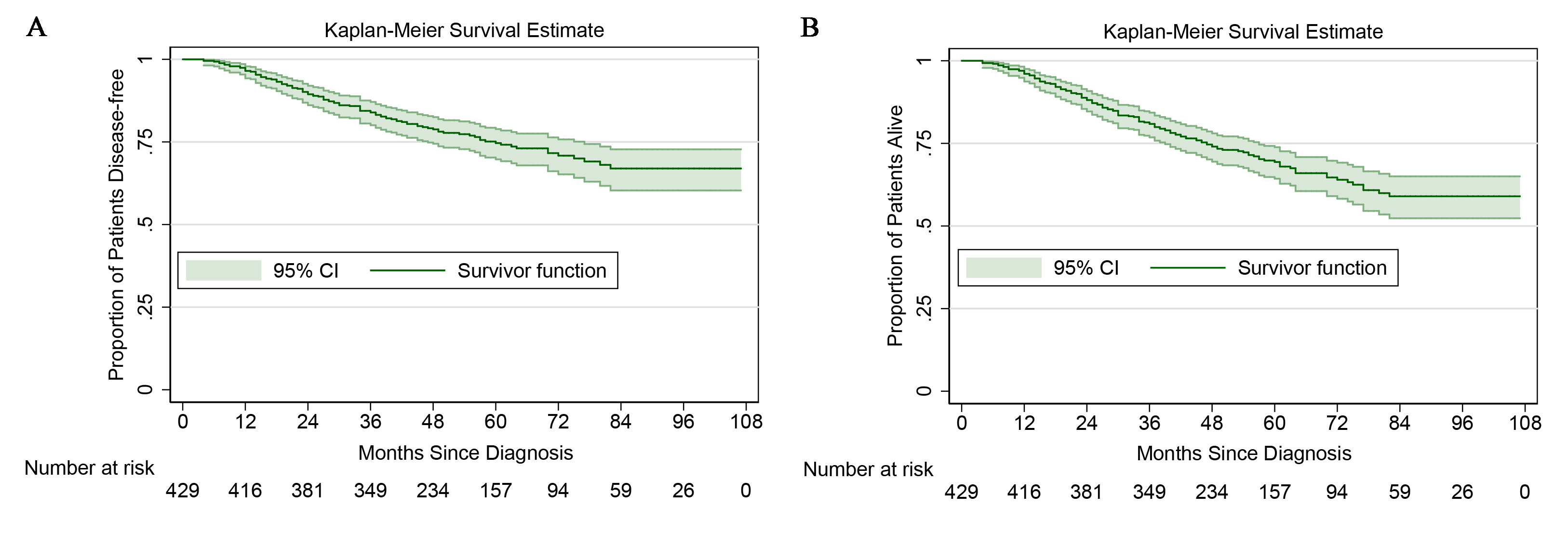

Supplement: Supplementary Material 1 — Kaplan-Meier curves of disease-free survival and overall survival for patients with apparent early-stage type II endometrial cancer. (A for disease-free survival; B for overall survival). [file DataSheet_1.zip › Supplementary Material 1.jpg]
